# Enhanced Mechanical and Antibacterial Properties of Nanocomposites Based on Poly(vinyl Alcohol) and Biopolymer-Derived Reduced Graphene Oxide

**DOI:** 10.3390/polym13040615

**Published:** 2021-02-18

**Authors:** Beom-Gon Cho, Shalik Ram Joshi, Seongjin Lee, Shin-Kwan Kim, Young-Bin Park, Gun-Ho Kim

**Affiliations:** Department of Mechanical Engineering, Ulsan National Institute of Science and Technology, UNIST-gil 50, Ulju-gun, Ulsan 44919, Korea; chogony1@unist.ac.kr (B.-G.C.); shalik@unist.ac.kr (S.R.J.); grant@unist.ac.kr (S.L.); skim@unist.ac.kr (S.-K.K.)

**Keywords:** nanocomposites, shellac, poly(vinyl alcohol), thermally reduced graphene oxide, mechanical properties, thermal stability, antibacterial activity

## Abstract

Functionalized graphene–polymer nanocomposites have gained significant attention for their enhanced mechanical, thermal, and antibacterial properties, but the requirement of multi-step processes or hazardous reducing agents to functionalize graphene limits their current applications. Here, we present a single-step synthesis of thermally reduced graphene oxide (TrGO) based on shellac, which is a low-cost biopolymer that can be employed to produce poly(vinyl alcohol) (PVA)/TrGO nanocomposites (PVA–TrGO). The concentration of TrGO varied from 0.1 to 2.0 wt.%, and the critical concentration of homogeneous TrGO dispersion was observed to be 1.5 wt.%, below which strong interfacial molecular interactions between the TrGO and the PVA matrix resulted in improved thermal and mechanical properties. At 1.5 wt.% filler loading, the tensile strength and modulus of the PVA–TrGO nanocomposite were increased by 98.7% and 97.4%, respectively, while the storage modulus was increased by 69%. Furthermore, the nanocomposite was 96% more effective in preventing bacterial colonization relative to the neat PVA matrix. The present findings indicate that TrGO can be considered a promising material for potential applications in biomedical devices.

## 1. Introduction

Poly(vinyl alcohol) (PVA) is a widely used commercial polymer owing to its high transparency, hydrophilicity, and adhesive properties [1,2,3,4]. Furthermore, its strong oxygen barrier capabilities and mechanical and biodegradable properties promote the application of PVA in fiber manufacturing, food packaging, and biomedicine [5,6,7,8,9]. However, the mechanical and electrical properties of PVA are negatively affected by the poor dewetting properties of this polymer [10,11], and its solubility, viscosity, and film strength may also differ depending on the degree of saponification [12]. To improve the aforementioned properties, various inorganic nanomaterials, such as clay [13], carbon nanotubes [14], and metal oxide nanoparticles [15], have been incorporated into a PVA matrix. However, poor dispersion and significant agglomeration of these nanofillers in the PVA matrix limit the improvement of the physical properties far below the expected level. Hence, it is essential to synthesize PVA nanocomposites with homogeneously dispersed fillers, which can significantly improve PVA physicochemical properties.

Owing to its high thermal, electrical, and mechanical properties, graphene has also been considered a potential filler for the synthesis of lightweight polymer nanocomposites [16]. However, the current manufacturing methods are not appropriate for producing high-quality graphene at the industrial scale [17]. Furthermore, owing to the presence of strong van der Waals forces, the obtained graphene flakes exhibit poor dispersion in the polymer matrix [18,19]. On the contrary, graphene oxide (GO) exhibits homogeneous dispersion properties due to the presence of oxygen-containing functional groups that establish strong interactions with the hydroxyl groups of the polymer matrix [20]. This homogeneous dispersion of GO at intermolecular level helps the efficient load transfer between the filler and the PVA matrix, resulting in significant enhancement of the physicochemical properties of the nanocomposites, even at low filler concentrations. However, GO is electrically insulating and thermally unstable; therefore, it needs to be reduced to restore the electrical and thermal properties [21]. During the preparation of polymer nanocomposites, the direct use of reduced graphene oxide (rGO) fillers is preferred to optimize the industrial synthesis and application of graphene-based polymer composites. However, the rGO chemical reduction process is time-consuming and requires the use of several toxic reagents that induce high structural disorder, which results in reduced mechanical properties of the polymer nanocomposites [22,23].

Shellac, a low-cost natural biopolymer, has been widely used in organic biomedical devices because of its excellent biocompatibility [24,25]. The long aliphatic carbon (C–C) backbone and a relatively lower thermal decomposition temperature of shellac provide an opportunity for more efficient graphitization than other synthetic polymers [26]. Earlier reports have shown that shellac-derived GO can be used as an active material in the fabrication of sensors and in photocatalytic applications [27,28,29]. Furthermore, it exhibits superior adhesion, due to the presence of oxygen-containing functional groups that form strong covalent bonds with the surface. This brought about a significant improvement in the interlaminar shear and flexural strength of GO [30]. These advantages of shellac-derived GO suggest its potential as a cost-effective polymer composite additive to improve the antibacterial and mechanical properties of materials.

The present study demonstrates the effect of various concentrations of shellac-derived thermally reduced graphene oxide (TrGO) on the thermal, mechanical, and antibacterial properties of PVA–TrGO nanocomposites. TrGO nanosheets were synthesized by a single-step thermal reduction process, in which homogeneous PVA–TrGO nanocomposites were produced by a water-based solution casting technique. The chemical and structural properties of PVA–TrGO nanocomposites were investigated using Fourier-transform infrared (FT-IR) spectroscopy and wide-angle X-ray diffraction (WAXD). Furthermore, the thermal, mechanical, and antibacterial properties of PVA–TrGO nanocomposites were investigated using differential scanning calorimetry (DSC), thermogravimetric analysis (TGA), tensile test, dynamic mechanical analysis (DMA), and Gram-negative *Escherichia coli* (ATCC 25922), respectively. It is expected that this study will help elucidate the effect of the nano-and microscale TrGO reinforcement of a PVA matrix and provide a viable method for the synthesis of polymer nanocomposites used in medical applications requiring improved mechanical and antibacterial properties.

## 2. Materials and Methods

### 2.1. Materials

A 99% hydrolyzed PVA (M_w_ = 31,000–50,000) was purchased from Sigma-Aldrich (St. Louis, MO, USA). Shellac flakes were purchased from Shellac Shack (Port Orford, OR, USA). Isopropanol (IPA; 99.5%) was purchased from Sigma-Aldrich (St. Louis, MO, USA).

### 2.2. Fabrication of PVA–TrGO Films

The PVA–TrGO nanocomposites were synthesized using a solution casting technique. Shellac was used as a carbon precursor to synthesize TrGO nanosheets via a single-step thermal reduction process. First, ball milling (planetary ball milling PM100 (Retsch), Hamburg, Germany) of shellac flakes (10 g) was carried out in a stainless-steel jar while agitating at a revolution speed of 200 rpm and an autorotation speed of 250 rpm. Ball milling running and cooling were performed for 5 min, to complete one milling cycle. A total of 30 milling cycles was performed, corresponding to a milling time of 150 min. Fourier Transform Infrared (FT-IR) spectra for shellac powder were obtained to observe any impurities during the ball milling process, as shown in Figure S1. The resultant powder was then placed into a tube furnace (Kejiafurnace, Zhengzhou, China) for thermal reduction. The ramping rate of the tube furnace was set to 3 °C/min and maintained at 900 °C for 30 min under a vacuum of 0.12 mbar.

**Figure 1:**
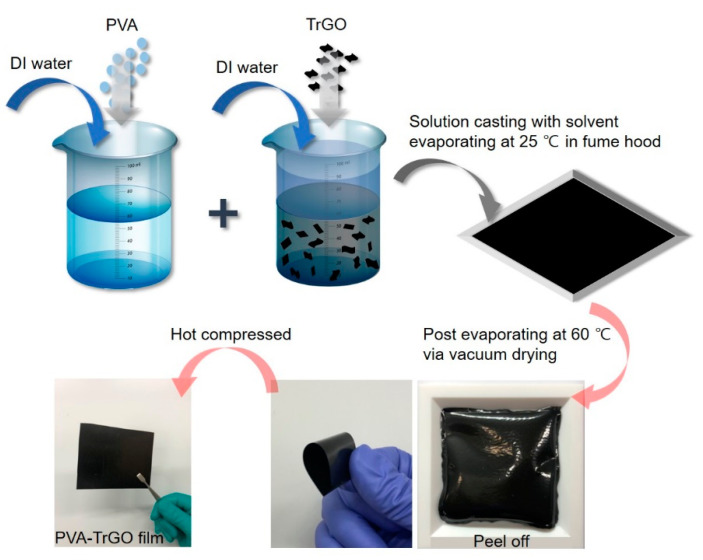
Schematic representation for the synthesis of poly(vinyl alcohol (PVA)–thermally reduced graphene oxide (TrGO) nanocomposite films.

PVA was dried at 90 °C for 24 h in a vacuum drying oven (Thermo Fisher Scientific, Waltham, MA, USA). Three grams of dried PVA was dissolved in 10 mL of distilled (DI) water at 90 °C at a stirring rate of 300 rpm. Various concentrations (0.1 wt.%, 0.5 wt.%, 1 wt.%, 1.5 wt.%, and 2 wt.%) of TrGO were dispersed in 10 mL of DI water by ultrasonication (Branson5800, Branson Ultrasonic Corp., Brookfield, CT, USA) for 1 h. The dispersion was placed in a homogenizer (T 18, IKA, Staufen, Germany) for 10 min, followed by a bath sonication process for another 60 min. The resultant TrGO suspension was then added to the PVA solution and mixed by bath sonication for 30 min. After homogenization, the mixture was poured into a Teflon mold and dried at 25 °C for 1 d in a fume hood (Nara fume hood, Flowmaster, Seoul, Korea) until solidified, as shown in Figure 1. The Teflon mold was kept at 60 °C in a vacuum oven (OV11, JEIO Tech, Daejeon, Korea) until the mass of the sample was constant. Finally, the resultant film was peeled off from the mold and compressed for 10 min using a hot press technique to eliminate voids. The resulting film surface was smooth and had an average thickness of 180 µm.

### 2.3. Characterization

#### 2.3.1. Thermally Reduced Graphene Oxide

Raman spectra of shellac-derived TrGO flakes were acquired in backscattering mode using an ultrahigh-throughput spectrometer (alpha300R, WiTec, Ulm, Germany). X-ray diffraction (XRD) measurements were performed using a Bruker D8 Advance diffractometer (Bruker, Billerica, MA, USA) with Cu Kα radiation (0.154 nm). The crystallinity and elemental mapping were investigated using high-resolution transmission electron microscopy (HR-TEM, JEOL Ltd., Tokyo, Japan) and high-angle annular dark-field scanning TEM (HAADF-STEM) fitted with an aberration corrector (CEOS GmbH, Heidelberg, Germany). A JEOL JEM-2100F electron microscope (JEOL Ltd., Tokyo, Japan) operating at 200 kV was used to perform the HR-TEM and HAADF-STEM. The chemical analysis was performed using X-ray photoelectron spectroscopy (XPS) (Thermo Fisher Scientific, Waltham, MA, USA). The system was equipped with a double-focusing hemispherical analyzer and a monochromatic Al Kα source (1486.6 eV) (Thermo Fisher Scientific, Waltham, MA, USA). The vacuum of the main chamber was maintained at 1 × 10^−10^ mbar during the entire measurement. XPSPeak41 software developed by Raymund Kwok was used for the deconvolution of the high-resolution XPS spectra.

#### 2.3.2. PVA–TrGO Nanocomposites

FTIR spectra of the PVA–TrGO nanocomposites were obtained using Varian 670-IR (Agilent Technologies, Santa Clara, CA, USA) to investigate the formation of hydrogen bonds between PVA and TrGO with different TrGO concentrations. The crystallinity of the nanocomposites was analyzed using wide-angle X-ray diffraction (WAXD). WAXD patterns of the PVA–TrGO nanocomposites were obtained in the 20 range 8–40° at a rate of 2°/min, using a high-resolution X-ray diffractometer (D8 Advance, Bruker, Billerica, MA, USA) with Cu Kα target. The crystallinity index (CI) of the film was calculated using the following equation:

(1) $$CI=\;\frac{A_{c}}{A_{c}+\;A_{a}}$$

where *A_c_* is the integrated area of the crystalline peaks, and *A_a_* is the integrated area under the amorphous halo. The thermal studies were conducted using DSC (Q200, TA Instruments, New Castle, DE, USA) and TGA (Q500, TA Instruments, New Castle, DE, USA). During the DSC measurement, nitrogen gas was purged into the chamber at a flow rate of 50 mL/min. The crystallization isotherms of the samples were investigated by completing a heating–cooling cycle where the samples were first heated from 30 °C to 250 °C at a ramping rate of 10 °C/min and then cooled to 0 °C at a ramping rate of 10 °C/min. The third scan was performed from 0 °C to 250 °C at a rate of 10 °C/min, during which the melting endotherms were identified. The degree of crystallinity (*Χ_c_*) was calculated as follows:

(2) $$X_{c}=\;\frac{\Delta H_{m}}{\Delta H_{m}^{0}}\times 100\%$$

where Δ*H_m_* and $\Delta H_{m}^{0}$ (138.7 J/g) are the enthalpies of the nanocomposite and pure PVA matrix, respectively. The thermal stability of the PVA–TrGO nanocomposites with varying TrGO concentrations was investigated using TGA in a nitrogen environment. The samples were annealed from 30 °C to 800 °C at a ramping rate of 10 °C/min. The morphologies and dispersion states of the nanocomposites were investigated by field-emission scanning electron microscopy (FE-SEM) (FEI Nova Nano 230, New York, NY, USA) at 10 kV accelerating voltage. Cross-sectional surface analysis was performed by fracturing the samples in liquid nitrogen and coating them in a platinum layer. The tensile properties of the nanocomposites, with sample dimensions of 5 mm × 30 mm × 0.18 mm, were analyzed using a universal test machine (UTM, Shimadzu, Kyoto, Japan) with a crosshead speed of 1 mm/min, in accordance with the ASTM D882 standard. The dynamic mechanical behavior of the composites was analyzed using dynamic mechanical analysis equipment (Q800, TA Instruments, DE, USA), in accordance with the ASTM D4065 standard. The measurements were carried out in multi-frequency strain mode using a tension film clamp within the temperature range of 40 °C–150 °C, at a heating rate of 3 °C/min and a frequency of 1 Hz.

The antibacterial activities of the nanocomposites were investigated using Gram-negative *E. coli* (ATCC 25922). The bacterial strain of *E. coli* (ATCC^®^ 25922™) from a stock was streaked onto a Luria–Bertani (LB) agar plate (Difco Generic LABWARE, Cadorago, Italy) and incubated at 37 °C with 5% CO_2_. After 18 h of incubation, the isolated colonies that appeared were inoculated into 10 mL of LB medium (Difco Generic LABWARE, Cadorago, Italy ), incubated at 37 °C, and stirred at 150 rpm until the logarithmic growth phase was achieved with a concentration in the range of 10^7^–10^8^ colony-forming units (CFU)/mL. Approximately 100 μL, at a concentration of 10^8^ CFU/mL, of the bacterial suspension was added to the surface of the PVA–TrGO nanocomposites. Bacterial cells were dispensed on empty Petri dishes as the control. After the treatment, the bacterial solution on the nanocomposite surface was successively diluted 10^1^–10^6^ fold in sterile phosphate-buffered saline (PBS) and cultured on LB agar plates. The cultured plates were incubated at 37 °C with 5% CO_2_ for 18 h. The experiment was performed in triplicate for accurate statistical analysis.

## 3. Results and Discussion

### 3.1. Structural and Chemical Characterization of Shellac-Derived TrGO

**Figure 2:**
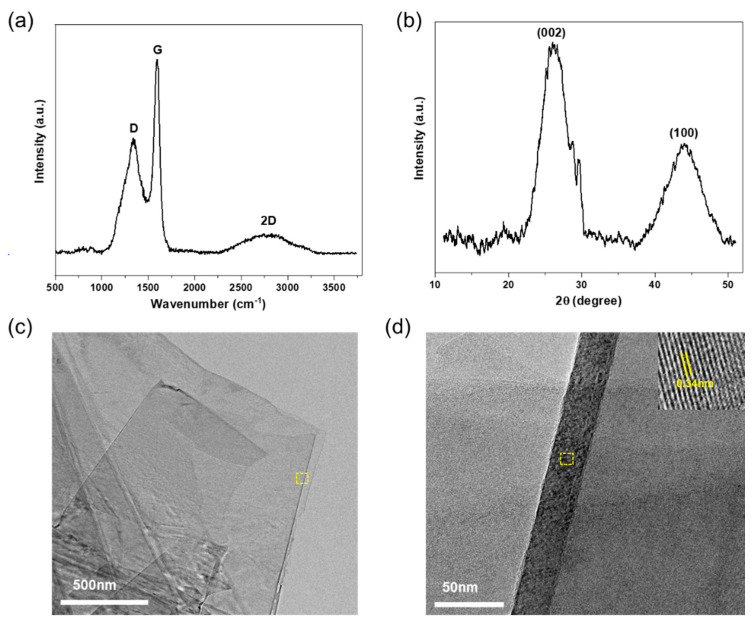
Structural characterization of TrGO synthesized from shellac using (**a**) Raman spectra and (**b**) x-ray diffraction spectra, (**c**) high-resolution TEM image (20,000×) of few-layered TrGO nanosheets, (**d**) magnified (150,000×) HR-TEM image to validate the crystallinity of TrGO.

A structural analysis of TrGO was performed using XRD and Raman spectroscopy, and the results are shown in Figure 2. The Raman spectra of TrGO displayed three main peaks: a D-band (~1345 cm^−1^), a G-band (~1592 cm^−1^), and a broad *2D* band (2300–3200 cm^−1^), consistent with previous reports [22,30,31]. It was observed that the intensity ratio of the *D* and *G* bands (I_D_/I_G_) was ~0.65, which was significantly lower than that previously reported for rGO [22,32]. It is hypothesized that the thermal reduction of TrGO resulted in lower structural disorder and an increase in in-plane crystallinity [33], whereby the disordered structure and defects therein were removed. Furthermore, the XRD spectrum of TrGO exhibited a diffraction peak at ~26.2°, which corresponds to the presence of (002) graphitic planes [34]. Using Bragg’s equation, the interplanar spacing (*d*) was estimated as ~0.334 nm, suggesting the efficient graphitization of the material. This result was clearly supported by HR-TEM, as illustrated in Figure 2c,d. As shown in Figure 2c, the TrGO nanosheets exhibited a smooth layered structure with a lateral dimension of ~2 µm. The average lattice spacing was estimated as ~0.34 nm (Figure 2d, inset), which is in close agreement with the interplanar spacing of the graphite lattice [33].

**Figure 3:**
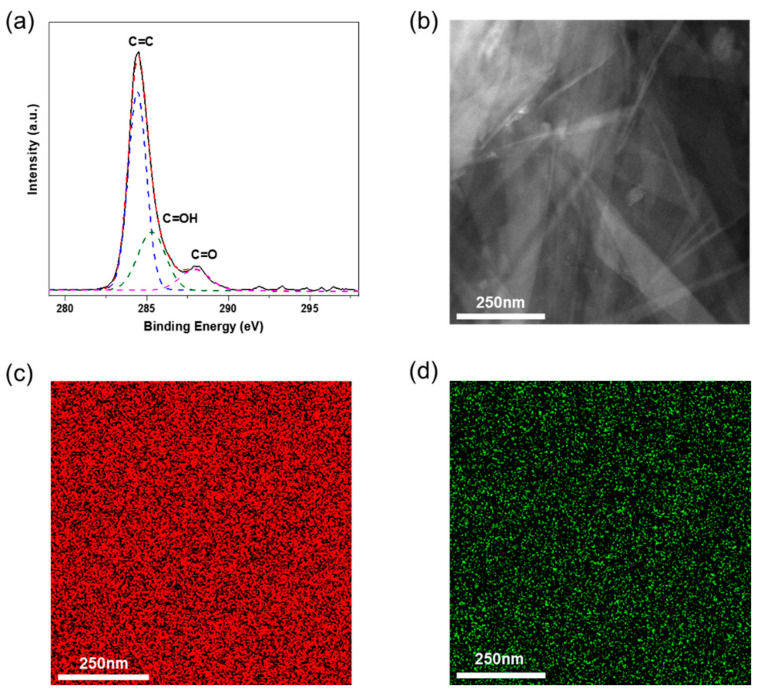
(**a**) High-resolution carbon (1s) core level XPS spectra, (**b**) HAADF-STEM image and elemental mapping exhibiting the distribution of (**c**) carbon (red) atoms and (**d**) oxygen (green) atoms of shellac-derived TrGO.

The high-resolution C−1s spectrum of TrGO was deconvoluted to investigate the presence of different oxygen-containing functional groups, and the results are shown in Figure 3a. The spectrum was deconvoluted into three main peaks, corresponding to C=C (sp^2^, 284.4 eV), C–OH (hydroxyl, 285.3 eV), and C=O (carbonyl, 287.9 eV) [35]. These oxygen-containing functional groups play a critical role in the homogeneous dispersion of TrGO in the PVA matrix, which affects the mechanical strength of PVA–TrGO nanocomposites and will be discussed later. As shown in Figure 3b–d, the elemental mapping of TrGO (C (red) and O (green)) revealed the presence of carbon and oxygen in shellac-derived TrGO.

### 3.2. PVA–TRGO Nanocomposites’ Properties

#### 3.2.1. Structural Analysis

**Figure 4:**
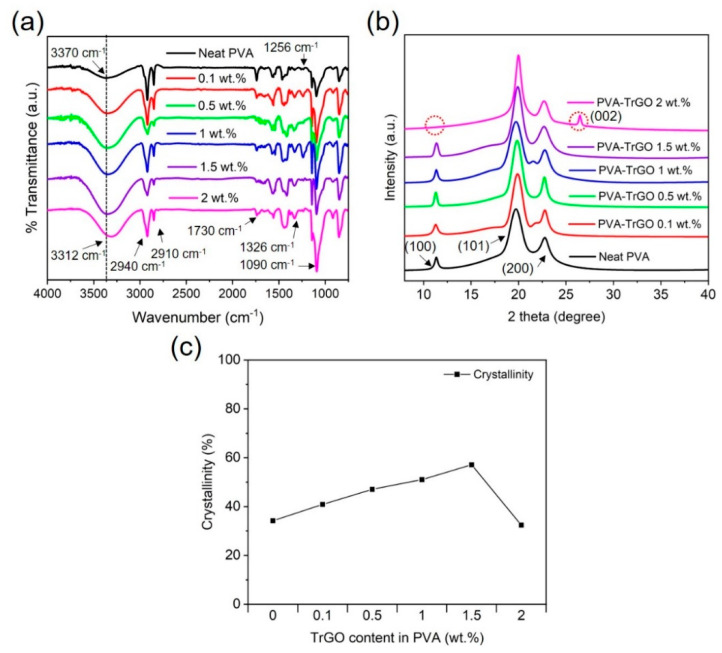
(**a**) FTIR spectra and (**b**) WAXD pattern for PVA–TrGO nanocomposites with different TrGO concentrations; black line: pure PVA matrix, red line: PVA–TrGO-0.1 wt.%, green line: PVA–TrGO-0.5 wt.%, blue line: PVA–TrGO-1 wt.%, purple line: PVA–TrGO-1.5 wt.%, pink line: PVA–TrGO-2 wt.%, (**c**) crystallinity of PVA–TrGO nanocomposites with different TrGO concentrations obtained from WAXD spectra.

The chemical structure analysis of the PVA–TrGO nanocomposites was performed using Fourier transform infrared (FTIR) spectra, and the results are presented in Figure 4a. The appearance of a broad band (3000–3500 cm^−1^) in the pure PVA matrix was attributed to the symmetrical stretching vibration of the hydroxyl group, which indicates strong intermolecular and intramolecular hydrogen bonding [36]. Other absorption peaks were assigned as follows: the peaks at 2940 cm^−^^1^ and 2910 cm^−1^ arose from asymmetric CH_2_ stretching [23,37], while those at 1730, 1326, 1256, and 1090 cm^−1^ were ascribed to C=O stretching, O–H bending, C–H bending, and C–O stretching vibrations, respectively, of PVA (Table 1). However, for the PVA–TrGO nanocomposites, the hydroxyl peaks systematically decreased (from 3370 cm^−1^ to 3312 cm^−1^) with increasing TrGO concentration, indicating the formation of hydrogen bonds between the hydroxyl groups of PVA and the oxygen-containing functional groups of TrGO. Furthermore, the peak intensity increased with increasing TrGO concentration up to 1.5 wt.%. The reduction in the peak intensity observed for the PVA–TrGO-2 wt.% sample can be attributed to the decrease in hydrogen bonds, which was caused by the agglomeration of TrGO sheets by strong van der Waals forces.

Figure 4b shows the WAXD patterns of the PVA–TrGO nanocomposites with different TrGO concentrations. For the pure PVA matrix, the WAXD patterns exhibited three major peaks at 19.7°, 22.6°, and 11.3°, which represent the (101), (200), and (100) planes, respectively [38,39,40]. As TrGO was introduced in PVA, the WAXD patterns showed diffraction peaks similar to those of pure PVA, which implies that the TrGO sheets were homogeneously dispersed in the PVA matrix. Interestingly, the WAXD diffraction pattern for the PVA–TrGO-2 wt.% sample exhibited the appearance and disappearance of the characteristic peak of TrGO (002) and pure PVA (100), respectively, as shown by the red dotted circle in Figure 4b. The development of TrGO peaks at such high concentrations can be attributed to the agglomeration of TrGO nanosheets in the PVA matrix because of the strong van der Waals interactions between the sheets. Still, the crystallinity of PVA was attributed to hydrogen bonding with its branching chains [41]. The stacking of polymer chains increases the WAXD peak intensity and has a strong influence on the crystallinity and physicochemical properties of polymer nanocomposites [36]. As shown in Figure 4c, the crystallinity of PVA–TrGO nanocomposites increased up to TrGO concentrations of 1.5 wt.%, which was due to increased intermolecular interactions between the filler and the matrix and resulted in enhanced mechanical properties. This phenomenon will be discussed later.

#### 3.2.2. Thermal Properties

**Figure 5:**
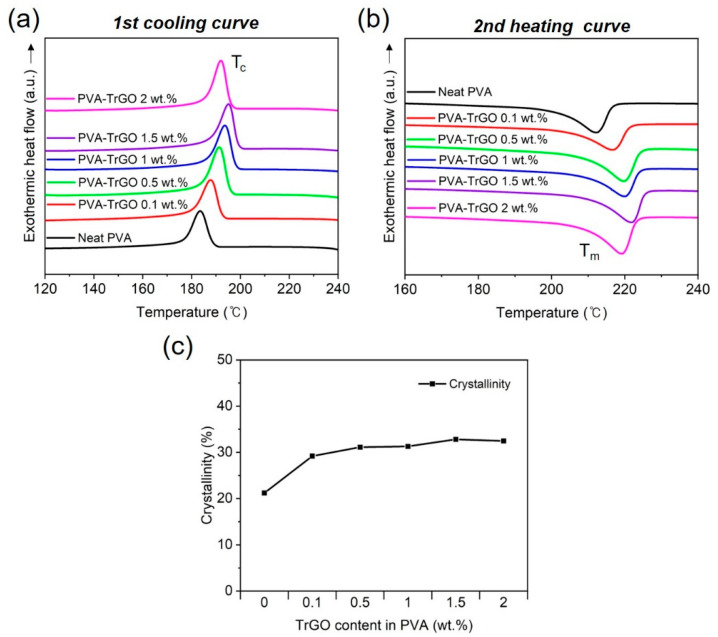
Non-isothermal DSC scans of (**a**) first cooling curves and (**b**) second heating curves of PVA–TrGO nanocomposites; (**c**) calculated crystallinity of the PVA–TrGO nanocomposites from DSC scans.

DSC analysis was conducted to investigate the effect of TrGO concentration on the thermal properties of the PVA–TrGO nanocomposites. The melting temperature (*T_m_*), crystallization temperature (*T_c_*), and degree of crystallinity (*%X_c_*) were calculated from the exothermic heat flow curve, as shown in Figure 5. As listed in Table 2, the *T_m_* and % *X_c_* values of the PVA–TrGO nanocomposites increased with increasing TrGO concentration up to 1.5 wt.%, which was due to the cumulative effect of heat-shielding capability and strong intermolecular interactions between the TrGO fillers and the polymer matrix [42,43,44,45]. Furthermore, the high surface-to-volume ratio and high surface energy of TrGO can also increase the crystallization and chain mobility of PVA [36]. It is worth mentioning that the discrepancy in the crystallinity fraction obtained from DSC and WAXD results has also been shown in previous reports [46,47,48]. Especially for PVA–TrGO-2wt.% nanocomposites, the deviation was quite significant, which can be attributed to the following factors: firstly, DSC provides information from the whole sample, whereas WAXD retrieves information within a few µm, hence is surface-sensitive. Thus, the inhomogeneous distribution of TrGO nanofillers in the PVA matrix can lead to a different crystallinity fraction, but with a similar trend [49], as observed here. Secondly, when the material undergoes thermal treatment during DSC measurement, the temperature elevation promotes relaxation, which can cause a partial structural recovery, leading to a deviation from the true crystallinity of the material, as observed using WAXD. Furthermore, the DSC technique affects the nonequilibrium state because of the inherent heating process, whereas the WAXD technique does not include any strong interaction with the measured system, and the sample is not subjected to any strong perturbation [48].

**Figure 6:**
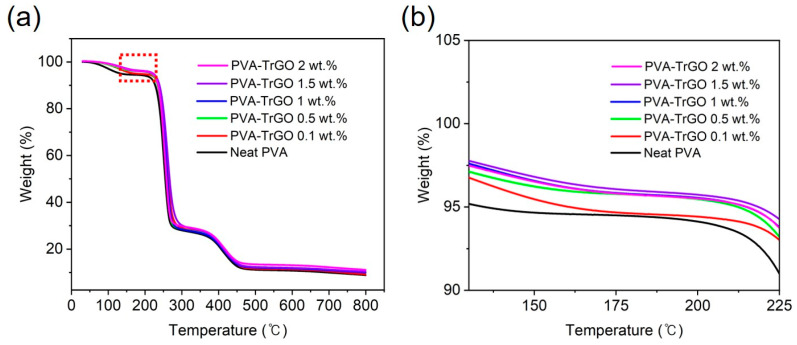
TGA thermographs of (**a**) PVA–TrGO nanocomposites with different TrGO concentration, (**b**) high-magnification region (130 °C–225 °C) displaying the 5% weight loss.

The thermal stability of PVA–TrGO nanocomposites was also measured using TGA, and the weight loss curves are shown in Figure 6a. As displayed in Table 3, up to a TrGO concentration of 1.5 wt.%, the degradation temperature at the weight loss of 5% increased gradually (Figure 6b) from 135 ℃ to 219 ℃. This indicated that the high heat resistance of TrGO contributes significantly to the thermal stability of PVA films [50]. However, for the TrGO-2 wt.% sample, it decreased to 215 ℃, which could be due to the high amount of TrGO in the PVA matrix causing agglomeration of the TrGO sheets, and thus, a decrease in heat resistance. The TrGO residue after thermal decomposition was found to increase gradually with increasing TrGO concentration.

#### 3.2.3. Morphology

**Figure 7:**
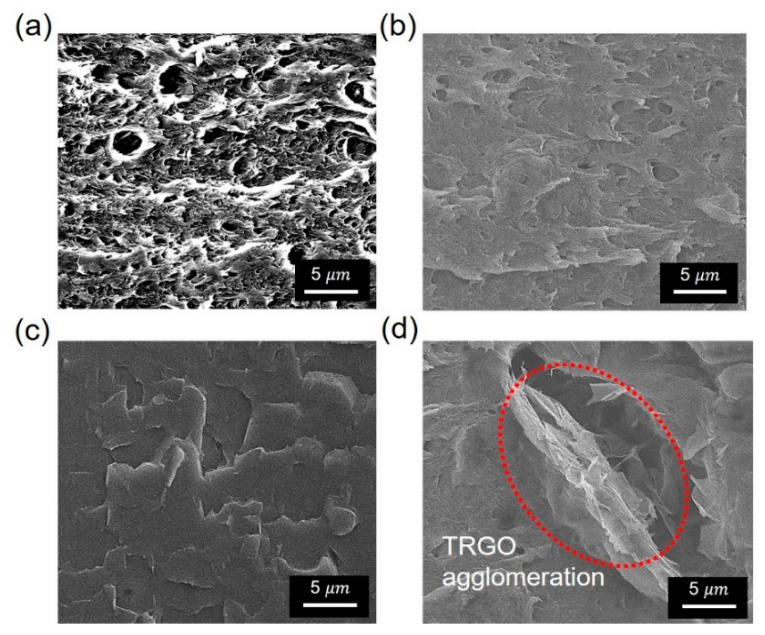
Cross-sectional SEM images (10000×) of fractured (**a**) pure PVA matrix and PVA–TrGO nanocomposites with TrGO concentrations of (**b**) 1 wt.%, (**c**) 1.5 wt.%, and (**d**) 2 wt.%.

The reported FE-SEM images (Figure 7) show the cross-sectional morphology of the fractured PVA–TrGO nanocomposites. For the pure PVA matrix, the surface appeared to be highly porous, as shown in Figure 7a. However, for PVA–TrGO-1 wt.% (Figure 7b), the surface exhibited a layered structure aligned in one particular direction and a notable roughness, as observed earlier [51]. Interestingly, for the PVA–TrGO-1.5 wt.% sample, the surface appeared to be smoother than that of the PVA–TrGO-1 wt.% sample. This might be due to the stable dispersion of TrGO without significant aggregation, leading to a smooth surface morphology. A homogeneous dispersion also improves the chemical affinity between TrGO and PVA, which increases the interfacial interaction, resulting in enhanced mechanical strength. However, when the TrGO concentration was increased to 2 wt.%, as shown in Figure 7d and Figure S2, local aggregation of the TrGO nanosheets in the PVA matrix could be observed because of the development of strong van der Waals attractive forces between the sheets due to the high TrGO concentration.

#### 3.2.4. Mechanical Properties

**Figure 8:**
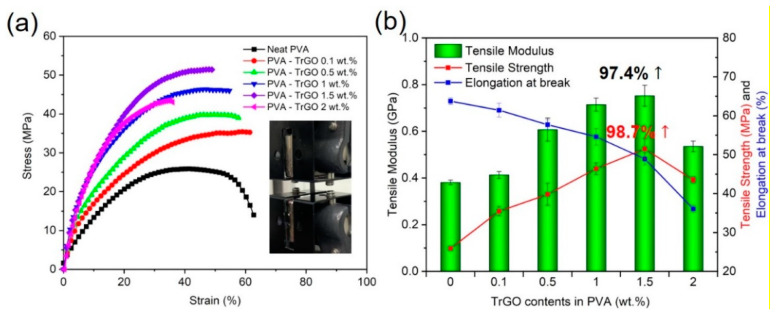
(**a**) Tensile stress-strain curves and (**b**) tensile strength, modulus, and elongation at break of PVA-TrGO nanocomposites with different TrGO concentrations.

**Figure 9:**
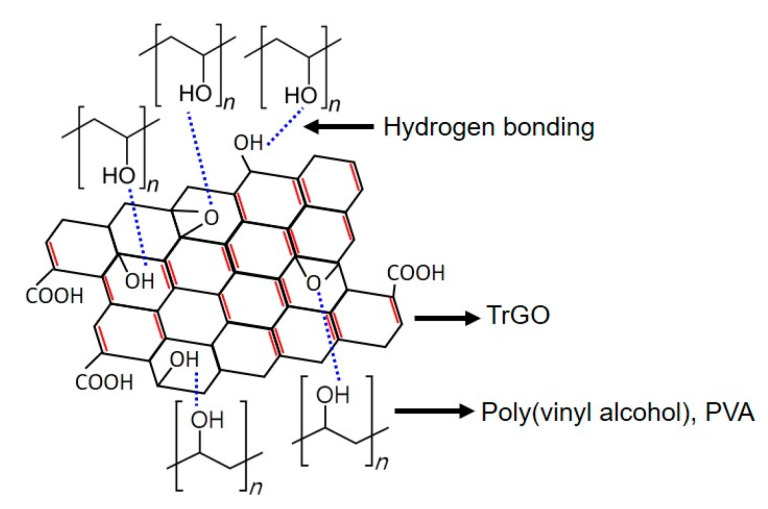
Schematic representation of the intermolecular interaction between the PVA chain and the shellac-derived TrGO.

**Figure 10:**
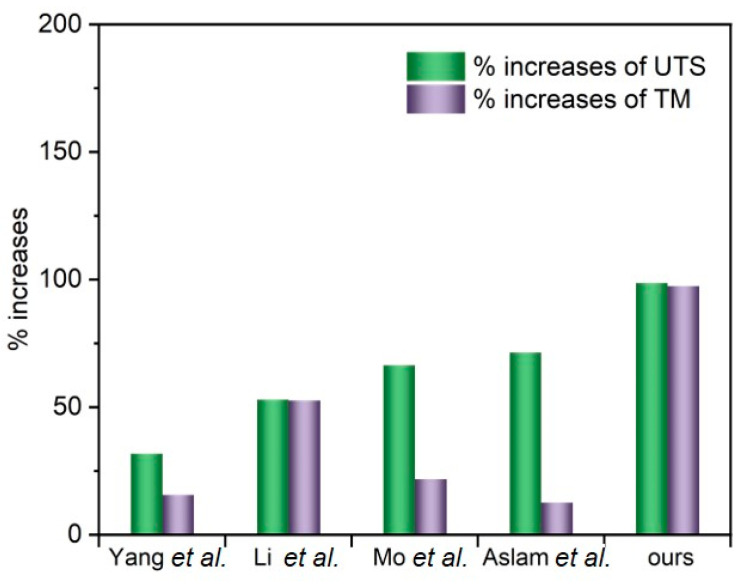
Comparison of percentage increases in the ultimate tensile strength (UTS) and tensile modulus (TM) of PVA–rGO composites with values reported in previous studies.

The tensile properties of the different PVA–TrGO nanocomposite samples were analyzed using a film tension test. As shown in Figure 8, the tensile strength and modulus of the PVA–TrGO-1.5 wt.% sample were ~51.5 MPa and ~750 MPa, respectively, which corresponds to ~98.7% and 97.4% increases, respectively, compared to those of the pure PVA matrix (~25.9 MPa and 380 MPa, respectively). The elongation at break decreased with an increasing TrGO concentration. It should be noted that the TrGO sheets are strong and rigid units, and the distribution of these units within the PVA matrix enhanced certain mechanical properties, such as stiffness and rigidity. This is because intermolecular interactions, such as hydrogen bonding between oxygen functional groups in TrGO and –OH groups in PVA, were induced, as shown in Figure 9. However, at the high concentration of 2 wt.% TrGO, the tensile properties deteriorated significantly because of TrGO agglomeration caused by van der Waal forces. As shown in Figure 10, the increase in the tensile properties of the presently investigated PVA–TrGO nanocomposites was compared with previously published results. Aslam et al. reported that addition of 0.02 wt.% of rGO to a PVA matrix enhanced the tensile strength and modulus by 71.4% and 12.5%, respectively, when compared to the control sample [52]. Mo et al. reported that addition of 10 wt.% of rGO in a PVA matrix enhanced the tensile strength and modulus by 66.4% and 21.7%, respectively [38]. Li et al. prepared PVA–rGO-3 wt.% nanocomposites by a solvothermal reduction process and reported a 53% and 52.6% increase in the tensile strength and modulus, respectively [53]. In addition, Yang et al. reported that the introduction of 3.5 wt.% graphene into a PVA matrix also improved the tensile strength and modulus by 31.8% and 15.5%, respectively [51]. However, in this study, tensile strength and modulus of the nanocomposite were improved by 98.7% and 97.4%, respectively, significantly more than what reported previously.

**Figure 11:**
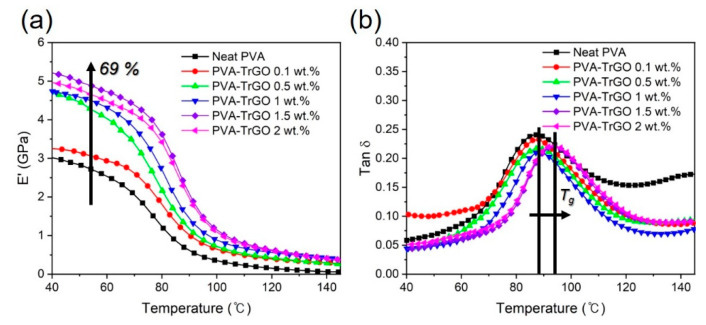
(**a**) Storage modulus (E′) and (**b**) tan δ of the PVA–TrGO nanocomposites with different TrGO concentrations.

The dynamic mechanical properties of the PVA–TrGO nanocomposites were used to investigate the elastic stiffness as a function of temperature. As shown in Figure 11a, the PVA–TrGO nanocomposites exhibited a systematic increase in the storage modulus (*E’*) with increasing TrGO concentration. In particular for the PVAy-TrGO-1.5 wt.% sample, *E’* increased to ~5.32 GPa, which represents a 69% improvement compared to that of the pure PVA matrix (3.15 GPa). The glass transition temperature (*T_g_*) was also determined from the peak temperature of the tan δ curves and is shown in Figure 11b. As expected, for the PVA–TrGO-1.5 wt.% sample *T_g_* increased to 93.6 ℃, which represents a ~7.4% improvement compared to that of the pure PVA matrix (87.2 ℃), which was ascribed to the strong attachment of TrGO to the pure PVA matrix, leading to increased rigidity (Figure 8b).

#### 3.2.5. Antibacterial Activity

**Figure 12:**
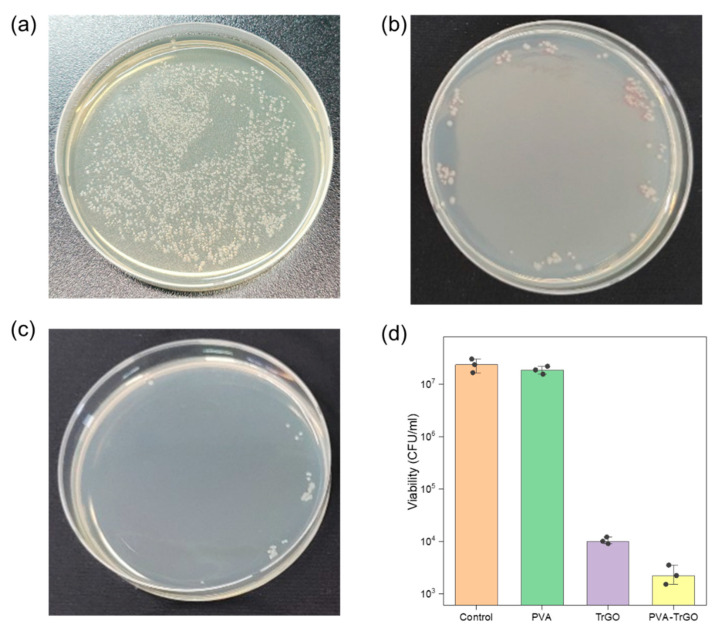
Antibacterial activity expressed as colony-forming units (CFU)/ml of (**a**) the pure PVA polymer matrix, (**b**) pure TrGO, and (**c**) PVA–TrGO 1.5wt % nanocomposite toward *Escherichia coli* (ATCC® 25922™) and (**d**) number of *E. coli* viable bacteria, calculated from CFU. Three independent measurements were performed for statistical analysis.

To investigate the potential of PVA-TrGO nanocomposites for biomedical applications, the antibacterial activity of the PVA–TrGO-1.5 wt % sample was studied. This sample was chosen because it exhibited the best thermal and mechanical properties among all samples. Pathogenic bacterial strains of Gram-negative *E. coli* (ATCC® 25922™) were used to evaluate the antibacterial activity by calculating the CFUs. Figure 12a–c shows the viability loss of the bacteria after 3 h of incubation with pure PVA, pure TrGO, and PVA–TrGO-1.5 wt %. Quantitative analysis based on CFUs, as illustrated in Figure 12d, indicated significantly reduced bacterial activity in the case of PVA–TrGO nanocomposites, showing an impressive ~3.5 log, which represents more than 96% reduction of *E. coli* viability, while the pure PVA matrix exhibited negligible antibacterial properties. The high antibacterial activity of the nanocomposites can be attributed to either of the following explanations: (a) direct contact of the bacteria with the edges of TrGO, which were able to pierce the cellular membrane, or (b) wrapping of TrGO around the bacteria thanks to its two-dimensional structure, which prevented nutrient uptake by the bacteria, resulting in cell death [54,55]. The relatively lower antibacterial activity of pure TrGO flakes with respect to the PVA–TrGO nanocomposite can be attributed to its strong tendency for aggregation due to the presence of strong van der Waals interactions between the nanosheets [56]. This restacking tendency of TrGO nanosheets causes the loss of its two-dimensional properties, leading to lower antibacterial activity with respect to homogeneously dispersed TrGO in a PVA matrix [57]. These results suggest that the PVA–TrGO nanocomposites, with superior mechanical and antibacterial properties, have the potential to become important materials in several biomedical applications.

## 4. Conclusions

In conclusion, shellac-derived TrGO was synthesized by a single-step thermal reduction process and exhibited homogeneous dispersion in a PVA matrix, allowing for simple solution casting to produce PVA–TrGO nanocomposites. The homogeneous dispersion of TrGO throughout the PVA matrix is attributed to the presence of strong hydrogen bonds between the hydroxyl groups of PVA and the oxygen-containing functional groups of shellac-derived TrGO, resulting in enhanced thermal and mechanical properties. In particular, in the samples containing 1.5 wt % TrGO, the tensile strength, tensile modulus, and storage modulus of the PVA–TrGO nanocomposites were increased by ~98.7%, ~97.4%, and ~69%, respectively. Furthermore, the nanocomposite also showed superior bactericidal performance, showing more than 96% antibacterial effectiveness towards *E. coli*, compared to the pure PVA matrix. These findings suggest that shellac-derived TrGO can be used as a reinforcement material for the synthesis of polymer nanocomposites for various biomedical applications.

## Figures and Tables

**Table 1:** Band assignments of PVA–TrGO nanocomposites obtained from the FTIR spectra.

Band (cm^−1^)	Assignments
1090	C–O Stretching, Epoxide Groups of PVA
1256	C–H Bending
1326	O–H Bending Vibration
1730	C=O Stretching
2910, 2940	Asymmetric CH_2_ Stretching
3000–3050	O–H Stretching

**Table 2:** DSC data and crystallinities of PVA–TrGO films with different TrGO concentrations.

TrGO Concentration (wt.%)	T_c_ (°C)	T_m_ (°C)	∆H_m_^a^	Χ_c_ (%) ^b^	CI (%) ^c^
0	183.43	212.23	29.44	21.23	34.2
0.1	187.65	216.77	40.5	29.2	40.89
0.5	191.36	219.78	43.15	31.11	47.04
1	193.56	219.95	43.39	31.28	51.04
1.5	195.14	221.91	45.51	32.81	57.1
2	192.06	219.23	45.03	32.47	32.42

**^a^** Enthalpies of the PVA–TrGO nanocomposites from DSC analysis software. **^b^** Calculated using Equation (2). **^c^** Calculated using Equation (1).

**Table 3:** Values of 5 wt.% loss temperature and percentage residue of PVA films at different TrGO concentrations.

TrGO Concentration (wt.%)	5% Weight Loss Temperature, T_D_^5%^ (°C)	Residual Amount at 800 °C (%)
0	134.75	6.78
0.1	160.89	7.51
0.5	212.31	7.61
1	214.86	7.69
1.5	218.54	7.83
2	215.09	8.83

## Data Availability

The data presented in this study are available on request from the corresponding author.

## Supplementary Materials

The following are available online at https://www.mdpi.com/2073-4360/13/4/615/s1. Figure S1: FT-IR spectra of shellac powder, Figure S2. FESEM (30000×) showing TrGO agglomeration in the PVA-TrGO-2wt.% nanocomposite (Red circular area in Figure 7d).
