# Supplementary material for: Enhanced Mechanical and Antibacterial Properties of Nanocomposites Based on Poly(vinyl Alcohol) and Biopolymer-Derived Reduced Graphene Oxide
Source: Polymers (Basel). 2021 Feb 18;13(4):615. doi: 10.3390/polym13040615 (PMC7923123; doi:10.3390/polym13040615)
Supplement: Supplementary file 1 [file polymers-13-00615-s001.pdf]

polymers

MDPI

Supporting Information

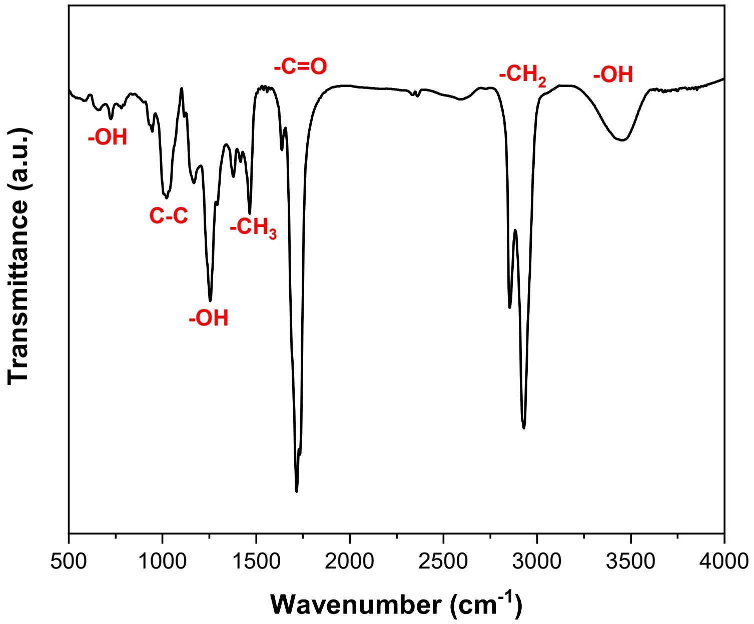

Figure S1. FT-IR spectra of shellac powder.

In order to investigate the chemical structure of shellac, FT IR spectroscopy was performed, and the results are shown in Figure S1. The FT-IR spectra of shellac used here display similar features as observed earlier [1, 2]. The appearance of a broad band between 3100 - 3700 cm^{-1} and 1250 cm^{-1} is due to the stretching and bending vibrations of the -OH group, respectively [3]. Other strong intensity peaks were assigned as follows: the peaks at 2855 cm^{-1} and 2935 cm^{-1} were observed due to the strong symmetric and asymmetric stretching vibrations of CH_2, respectively, peaks at 1712 cm^{-1} and 1733 cm^{-1} can be attributed to the C=O stretching vibrations of esters and acids, respectively [2, 4]. The weak bands of the shellac polymers are: peak at 1636 cm^{-1} (C=C stretching vibration), 1412 cm^{-1} (-CH_2), 1175 cm^{-1} (C-O stretching), 1050 cm^{-1} (C-C stretching), 1465 cm^{-1} and 1376 cm^{-1} were mainly due to the CH_3 asymmetric and symmetric bending, respectively [2, 5]. The weak signals at low wavenumbers can be attributed to the -OH out of plane deformation of carboxylic acids or aldehydes [2, 5].

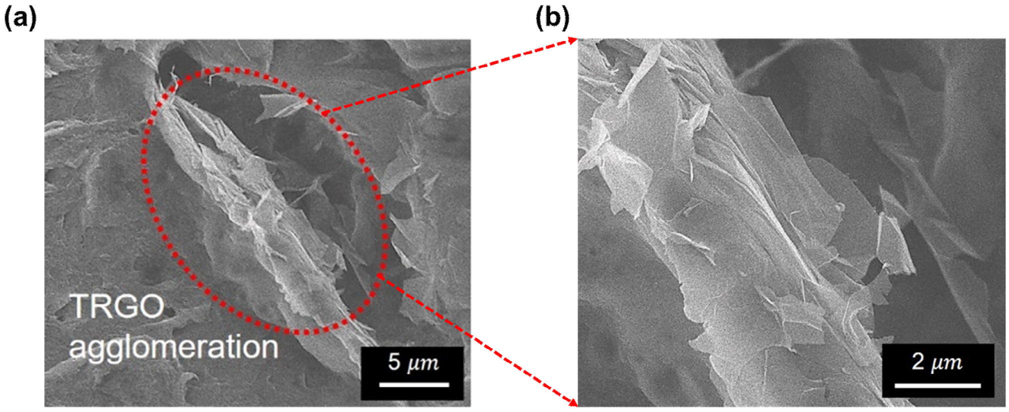

Figure S2. (a) Cross-section (10000x) and (b) zoomed-in (30000x) SEM image for PVA-TrGO-2wt.% showing TrGO agglomeration in the PVA matrix.

References

[1] S. Brajnicov, A. Bercea, V. Marascu, A. Matei, and B. Mitu, Shellac Thin Films Obtained by Matrix-Assisted Pulsed Laser Evaporation (MAPLE), Coatings 8(8) (2018).

[2] K. Li, H. Zheng, H. Zhang, W. W. Zhang, K. Li, and J. Xu, A novel approach to the fabrication of bleached shellac by a totally chlorine-free (TCF) bleaching method, RSC Advances 6(60) (2016) 55618-55625.

[3] M. Aslam, M. A. Kalyar, and Z. A. Raza, Polyvinyl alcohol: A review of research status and use of polyvinyl alcohol based nanocomposites, Polymer Engineering & Science 58(12) (2018) 2119-2132.

[4] V. Ravi, T. M. P. Kumar, and S. Ramaiah, Novel colon targeted drug delivery system using natural polymers, Indian J. Pharm. Sci. 70 (2008) 3.

[5] J. Derry, Investigating Shellac: Documenting the Process, Defining the Product. A study on the processing methods of Shellac, and the analysis of selected physical and chemical characteristics. (2012).
